# Pluripotent stem cells for pathological modelling of Hutchinson-Gilford Progeria Syndrome (HGPS) and drug discovery

**DOI:** 10.1186/1750-1172-10-S2-O2

**Published:** 2015-11-11

**Authors:** Xavier Nissan

**Affiliations:** 1 CECS, I-STEM, AFM, Institute for Stem cell Therapy and Exploration of Monogenic diseases, Evry, France

## 

Progeria, also known as HGPS, is a rare, fatal genetic disease characterized by an appearance of accelerated aging in children. This syndrome is typically caused by mutations in codon 608 (p.G608G) of the *LMNA* leading to the production of a mutated form of Lamin A precursor called progerin. In HGPS, progerin accumulates in cells causing progressive molecular defects including nuclear shape abnormalities, chromatin disorganization, DNA damages and delay in cell proliferation. Although two clinical trials have recently produced promising results, as well as *in vitro* and *in vivo*, there is currently no cure for HGPS patients. In collaboration with the teams of Dr Nicolas Lévy (*UMR_S 910*) and Dr Lino Ferreira (*University of Coimbra*), we have addressed this challenge by developing two high throughput screenings using the unique self-renewal and pluripotency properties induced pluripotent stem cells (iPS cells). Accordingly, these studies revealed the potential therapeutic effect of two new classes of compounds rescuing both nuclear shape abnormalities and defects of differentiation through on one hand, an inhibition of the prenylation process and on the other hand, a decrease of progerin expression.

